# Communicating during care transitions for older hip fracture patients: family caregiver and health care provider's perspectives

**DOI:** 10.5334/ijic.1076

**Published:** 2013-10-31

**Authors:** Christine Glenny, Paul Stolee, Linda Sheiban, Susan Jaglal

**Affiliations:** Department of Medicine, University of Toronto, 27 King's College Circle, Toronto, ON M5S 1A1, Canada; School of Public Health and Health Systems, University of Waterloo, 200 University Avenue West, Waterloo, ON N2L 3G1, Canada; School of Public Health and Health Systems, University of Waterloo, 200 University Avenue West, Waterloo, ON N2L 3G1, Canada; Department of Physical Therapy, University of Toronto, 160-500 University Avenue, Toronto, ON M5S 1A8, Canada

**Keywords:** care transitions, caregivers, hip fracture, communication

## Abstract

**Introduction:**

Older hip fracture patients frequently require care across a variety of settings, from multiple individuals, including their family caregivers. We explored issues related to information sharing during transitional care for older hip fracture patients through the perspectives of both health care providers and family caregivers.

**Methods:**

Thirty-five semi-structured interviews were conducted with family caregivers (n = 9) and health care providers (n = 26) of six hip fracture patients to gather perspectives on information sharing at each care transition, beginning with post-surgical discharge from acute care. Data were analysed using conventional qualitative content analysis methods using NVivo8 software.

**Results:**

Both family caregivers and health care providers recognise that family caregivers' involvement has important benefits for patients, but this involvement is frequently limited by poor information sharing. Barriers include limited staff time, patient privacy regulations and lack of a clear structure to guide information sharing. Receiving, not offering, information was the focus of information sharing by both family caregivers and health care providers.

**Conclusions:**

Specific barriers that lead to poor information sharing between family caregivers and health care providers have been identified in this study. Possible interventions to improve information sharing include encouraging communication with family caregivers as standard care practice, educational strategies and more effective use of health information systems and technologies.

## Introduction

Due to the complexity of their health conditions, older patients frequently require care from multiple individuals across a variety of settings [1–4]. ‘Care transition’ is a term used to describe a patient's movement from one health care setting to the next [5]. Ma and colleagues [6] found up to 80% of newly hospitalised elderly patients experience two to six care transitions within one year. Care transitions have been recognised as a high risk time for older adults [5] that can lead to fragmented care and negative outcomes, as well as greater use of hospital, emergency, post-acute and ambulatory services when managed poorly [4,7]. Despite the frequency and the importance of transitions, transitional care has until recently received little attention in research, health policy and clinical practice [4]. Earlier studies have tended to concentrate on a single move from hospital to home [8–12] which neglects a large variety of transitions across the entire continuum of care. There is also limited research that focuses on care transitions for more acutely ill elderly patients [13,14]. For these patients, the circle of care is often formed quickly due to the unexpected medical crises and composed of a variety of health care providers and family caregivers. Current recommendations highlight the need to address quality gaps occurring during transitional care and points to the need for better communication and more involvement of patients and family caregivers at all steps of the process [15–17].

Communication among health care providers is central to achieving successful care transitions [18–21]. However, communication and information sharing are often major challenges particularly for patients that require multidisciplinary care [22–25]. Previously, our team found that health care providers acknowledged the importance of expanding the circle of care for older patients with complex illnesses; though they also recognised that this often leads to a reduction in care continuity as a result of challenges with information sharing within and between settings [26].

Informal caregivers, often family members, frequently participate in caring for their elderly loved ones. The literature supports that family caregivers who receive adequate information and feel involved in hospital to home transitions are more satisfied, better accepting of their caregiving role and experience less anxiety than family caregivers who are not well informed [27–30]. Better communication with family caregivers during discharge from hospital has also been linked to increased patient satisfaction, fewer medical complications post-discharge, decreased rehospitalisations and lower cost to the health care system [28,30–32].

With growing emphasis on collaboration within a patient's circle of care over the past decade and with the significant increase in post-discharge care being provided by family caregivers, health care providers have been urged to develop ‘partnerships’ with family caregivers in hospitals, nursing homes and the community [33–37]. Despite the strong support for family centred care and recognition of the vital role of family caregivers during transitions, there is consistent evidence recognising a lack of communication between health care providers and family caregivers [17,28,38,39]. During times of transition, family caregivers report limited preparation for caregiving [25,40–42], insufficient information about managing the medical and psychological aspects of caring [17,32,43,44], lack of information related to resources and services in the community [2], difficulties managing and negotiating care with multiple providers [2], breakdowns in the transfer of information from health care providers to families (e.g. medication plans) [32,40,45–47] and communication issues between health care providers in different settings [42].

Hip fracture patients are an ideal model for understanding the experience of caring for the frail elderly during care transitions as they are very common in this population [48], and proper care for these patients requires management by multiple health professionals across multiple settings [1]. Hip fractures are most commonly seen in older adults, typically over the age of 65 and are a main cause of hospitalisation [48]. Between 2001 and 2005, there were a total of 131,350 hip fractures, in those ages 65 years or older [49]. In Ontario, Community Care Access Center case managers coordinate acute and long-term care services [50]. These case managers assist patients during transitions and who require the use of various health care services [50,51]. The aim of this study is to explore experiences related to communication and information sharing during in-hospital and transitional care for older hip fracture patients through the perspectives of both health care providers and family caregivers in order to identify areas to target future interventions. Unlike much of the previous work in this area, we interviewed both health care providers and family caregivers while they were actively caring for the same patient in order to illustrate their unique perspectives based on a linked experience [34,52–54].

## Methods

### Ethnographic field study

This investigation was conducted as part of a larger multi-site ethnographic field study called InfoRehab Transitions (www.inforehab.uwaterloo.ca). Our pan-Canadian team centred in British Columbia and Ontario focused on post-operative hip fracture care transitions for older patients. This study focused specifically on research conducted by the Waterloo, Ontario team. Our approach was guided by the principles of a focused ethnography, which include shorter-term field visits, larger amounts of data collection and intense analyses of interviews [55]. Ethnography is an ideal approach for health system research as it accounts for the organisational contexts of care and/or the extent to which the context itself may be contributing to problems [56]. Sensitising concepts were determined prior to data collection by each site to understand the assumptions and knowledge with which the researchers would understand and interpret the data [57]. These included the following:

- Older persons with hip fracture are exemplars of a typical frail older adult with multiple co-morbidities
- High demand for acute beds may lead to fast paced, poorly executed transitions
- All stakeholders involved in the care transition experience challenges
- Researchers are more familiar with challenges faced by health care providers

### Participants

The data used for this analysis include interviews with health care providers and family caregivers that were conducted in association with the University of Waterloo study centre in Ontario, Canada. As hip fracture patients transition across different health care settings and providers, the patient is the only constant variable throughout the entire trajectory of their recovery. For this reason, we initiated recruitment with the patient and coordinated our data collection process based on their care trajectory and changing circle of care. Patients were initially recruited post-surgery in an acute care setting. The inclusion criteria for patients consisted of: diagnosis of a hip fracture, ability to speak English and age greater than 65 years. Patients with moderate to severe cognitive impairment were excluded from this study. For additional information on our patient recruitment strategies, refer to Toscan and colleagues [58]. After the patient formally consented to participate in the study, we proceeded to recruit members of the patient's care network, including family caregivers and health care providers. Using a purposive sampling approach [59], we aimed to recruit at least two health care providers in each setting across the patient's care trajectory. We approached health care providers based on their level of involvement with the patient during their transition and how acquainted they were with the patient. We approached family caregivers based on cues from the patient (e.g. primary contact), and these individuals were also required to identify themselves as a family caregiver for the patient. In previous studies on transitional care, the perspectives of family caregivers and patients are often combined [28,60,61]. Though we acknowledge the patient as being at the centre of care and their role as essential in care transitions, in order to maintain focus on those providing care, we decided to limit our analyses to family caregivers and health care providers perspectives.

### Data collection

Two trained data collectors conducted the one-on-one in-person interviews with each participant. These interviews were completed following each care transition. For example, following the patient's discharge from acute care to inpatient rehabilitation, we aimed to interview two health care providers from the acute care setting regarding the patients' discharge, two health care providers from inpatient rehabilitation regarding the patients' admission and one family caregiver regarding the entire transition. This process was then repeated each time the patient transitioned to a new setting. The objective of these interviews was to understand transitional care, with a main focus on information sharing. The interviews were facilitated using semi-structured interview guides that consisted of pre-planned questions to assist in reminding the data collectors of the main topics that should be covered, as well as flexible probes created to promote natural conversations and generate additional in-depth discussion. Separate guides were developed for the health care providers and family caregivers to accommodate their unique perspective and level of knowledge. Questionnaire development was informed by a series of pre-field work interviews with health care providers [26]. Sample interview questions from each guide are provided in Table 1.

Data collectors also documented observations every 30 minutes throughout the study process. Field notes were taken from the time of entering the study sites and during the interview process. Verbal and non-verbal interactions between health care providers and family caregivers were also documented and field notes recorded on the environment in which these observations took place. The personal feelings of the data collectors were included in the observations, which assisted to further understand how personal emotions influenced how the data was interpreted [55].

### Data analysis

All interviews were audio-recorded and transcribed verbatim. The data were analysed using a qualitative conventional content analysis technique defined by Hsieh and Shannon [62] and Nvivo 8 qualitative software [63]. The interviews were initially read through multiple times by two members of the research team in order to achieve immersion. Each researcher independently read through the interviews and highlighted any discussions about information exchange. These highlighted components of each set of interviews were then coded and categorised by topic. To ensure consistency in the coding strategy, ‘inter-coder’ agreement was established by cross-checking the independently coded transcripts [59,64]. Any inconsistencies about the categorisations were discussed until agreement was reached between both researchers. This discussion was then followed by a collaborative approach where team members discussed the final codes and themes to reach consensus [64].

All of the interviews were conducted between January and December 2010 in the Waterloo-Wellington Region of Ontario. We recruited six patient care networks, which resulted in 26 interviews with health care providers and 10 interviews with 6 family caregivers. Table 2 gives a summary of the number of interviews completed in each care network.

All six family caregivers interviewed were females and ranged between the ages of 40 and 70 years. Five caregivers were children of the patient, and one was a spouse. Interviews with health care providers were conducted in seven settings along the continuum of care for hip fracture patients. Table 3 lists the care setting, role, and number of each type of health care providers. The majority of health care providers were female, and their years of experience in their current role varied from 4 months to 40 years. Due to poor patient health, one care network was classified as ‘loss to follow-up’ after completing the first transition. The data collected during the first transition (acute care to inpatient rehabilitation) were included in the analysis.

The study received ethics clearance through the Office of Research Ethics at the University of Waterloo, the Tri-Hospital Research Ethics Board, Community Care Access Centre and other relevant facilities as necessary.

## Results

Two themes were identified that related to communication and information sharing between health care providers and family caregivers for older hip fracture patients (Table 4). This first theme describes family caregivers and health care providers' current attitude towards communicating with each other, and the second theme identifies specific barriers that negatively influenced information sharing between these groups.

### Family caregivers and health care providers' current attitudes towards communicating with each other

#### Family caregivers and health care providers recognise caregivers' involvement is beneficial

The health care providers and family caregivers acknowledged that family caregivers have an essential role in transitional care for elderly patients. When the health care providers were asked what factors had a positive influence on care transitions, they often listed supportive family caregivers as an important component. The health care providers frequently reflected on the emotional support that family caregivers provide during transitions. One Registered Nurse described that:

It is always better to have a family member because then it makes the transition nicer for the person. There is a lot of nerves and a lot of uncertainty and you know, people are scared because they have gotten used to being here and the routine, and then do go on to another level. It is scary so family members are always encouraged to come. (Inpatient rehabilitation, nurse)

The health care providers acknowledged that having active family caregivers results in additional discharge options for the patient. They also reflected on the health system benefits that result from family caregivers being involved in transitional care including shorter hospital stay and fewer community home care hours.

The family caregivers were also aware of the important role they play in caring for the patient while they are in hospital and in the community. They perceived themselves as ‘glue’, ‘support’, and ‘leaders’ when they described their responsibilities. They also reflected on providing information to health care providers to help them care for the patient related to their history and background. One family caregiver recounted how she helped a physiotherapist communicate with the patient.

[The physiotherapist] wanted to get a history and know you know what type of life he had and because [the physiotherapist] said ‘I get the sense that he's stubborn and he really doesn't hear everything because all he keeps saying is I know, I know, yup, yup'…So I instructed them that you have to stand on his left, he has a hearing aid, you have to speak loudly and clearly and he should hear you then. (Family Caregiver)

The family caregivers also described that they facilitated information sharing between health care providers. They often provided names and contact information to health care providers as well as relay verbal messages and written documents between settings.

#### Family caregivers actively seek information from health care providers

The family caregivers and health care providers both described that family caregivers took responsibility for initiating contact with the health care providers. Most often the health care providers described wanting to spend time with the patient, gathering information and typically would only contact the family caregivers if they needed them to communicate with the patient or perform a specific task. The health care providers expressed that it was appropriate, and they expected to wait for family caregivers to contact them.

I didn't speak with the daughters. They didn't contact me. If they want any information I'd wait for them to ask for it. (Acute care, Inhospital CCAC case manager)

Many of the family caregivers felt surprised and confused when the health care providers did not contact them early during the patient's admission. The family caregivers felt uninformed and compelled to contact the health care providers in order to initiate communication.

I am the one who called actually, made contact with her because I wanted to figure out what was going on. (Family Caregiver)

The family caregivers described that it was necessary for them to be persistent and proactive in order to collect information from the health care providers. Researchers observed family members waiting and discussing the frustrations around not having an update on the patient's status. Family caregivers frequently indicated that they were required to independently identify and learn this proactive behaviour while they were interacting with the health care providers.

Once I knew what and where to go I just waited until I got the information, like I wouldn't go away, just keep on asking for it and I did get it. (Family Caregiver)

I just put my foot down and said I need this, this, this. So when you're asking, yes, I did put my foot down. So wanted a specific answer and I got them, I put my foot down. (Family Caregiver)

The family caregivers were also responsible for asking questions to prompt the health care providers to share information with them. Some family caregivers described not knowing what questions to ask to get the information they needed. Others noted that it was challenging for family caregivers to know what information they required:

I was the one asking the questions and I think that helped, but I can see that with somebody without the articulation it could be difficult for them to even understand what's needed, what's required. (Family Caregiver)

#### Receiving information is portrayed as the focus of information sharing

When health care providers and family caregivers described why and how they communicate with each other, both groups focused on gathering information from the other party. The health care providers focused on receiving information from the family caregivers about the patient and previous health care settings. They discussed the benefits of collecting information from the family caregivers to verify the patients' true health status.

The family did have concerns about her personal safety when she was at home. So knowing that information is really important when deciding discharge location…they see what's really going on to give you a lot of really good information about what is realistic and what is appropriate. (Inpatient rehabilitation, occupational therapist)

The individual themselves they're worried that they're going to be put in a nursing home, that's their biggest fear. ‘I'd rather die than be put in a nursing home’. So many times they will underplay the difficulties that they're having and it's not until other family members come by - ‘oh she did have two falls yesterday’.. . So I rely a lot on families stories. (Retirement home manager)

Many health care providers discussed that it was only important for them to contact the family caregivers when the patient was either a poor historian or not well oriented. In these cases, the health care providers recognised that they required information from the family caregivers.

If the resident is very much oriented, totally oriented than we will, basically I don't talk to the kids and kin. (Home care, physiotherapist)

I found him very capable, aware, he didn't sound confused in any way or you know or had any type of dementia, there was no history of dementia that I saw from the notes. So I had really no reason to speak to his family members. (Home care, community CCAC case manager)

The family caregivers also described themselves as mainly receiving information from health care providers. Though they were aware that they had important information to share with health care providers regarding the patients' previous health status, they felt that the health care providers had a more inclusive and educated understanding of the patients' current condition. Overall, neither the family caregivers nor the health care providers described themselves as a source of information or focused on providing information to each other.

### Specific barriers that negatively influences information sharing between these groups

#### Health system issues challenge information sharing

Family caregivers and health care providers both perceived time as a major barrier to information sharing. The health care providers described that they were willing to share information with family caregivers; however, due to their other responsibilities - especially documentation - they did not have time to speak with them. One nurse in inpatient rehabilitation said:

It's just people and families are getting more frustrated and you need to spend more time and we don't have that. We live in a society where we have to document everything for the college because we're protecting ourselves without having all these fines and if we didn't have to do that … we need to be more vocal and not be so rushed. (Inpatient rehabilitation, nurse)

The family caregivers were also aware, based upon their observations on the units, that health care providers were often too busy with their other tasks to share information with them. The researchers observed a fast-paced environment of health care providers within the health care setting. There were many staff shift changes observed, as well as actual movement of staff between different sections of the health facility, which influenced the continuity of care with patients during a shift or during the week. The family caregivers often expressed sympathy for the health care providers when they referred to the demanding schedule.

I find the nurses are very, very nice. They try their best but you can see them running around too much and if there was an aide to look after people's needs that would be a different matter. But they themselves are always running around. (Family Caregiver)

Some family caregivers expressed feeling guilty when they approached health care providers to communicate with them. They described feeling that they should not be ‘taking up their time’ or ‘preventing them from caring for patients’.

The researchers observed and heard family members expressing their concerns about the plan for discharge, where the patient was going and when they may be transitioning. Both family caregivers and health care providers described that moving between settings often occurred too rapidly and without warning.

It's the typical indecision day by day thing and that's what sometimes the family can't get the concept of and you know he's doing really, really poor but things ultimately start to clear whether it's medications or anesthetic or you know whatever. Then they're good to go and there's no holding it back - you just go. (Acute care, Inhospital CCAC case manager)

It was a surprise…just that it all happened all of a sudden and they didn't want to lose the bed and they just brought him here. (Family Caregiver)

Both groups also recognised that the short time interval between making the decision for a patient to be transferred and the actual move made it difficult to communicate with each other. Overall, the health care providers accepted this situation as an unfortunate and unavoidable reality of institutional care; while the family caregivers perceived this process as being disorganised and lacking in patient and family involvement.

Patient privacy regulations also had an effect on communication between health care providers and family caregivers. Health care providers described that their most important matter to consider when deciding what information to share with the family caregivers was patient privacy. The health care providers explained that they required direct permission from the patient before they were able to share information with the family caregivers.

With her permission, if I do contact her family I have to get her permission to call them and let her know what I'm going to tell them. As it is appropriate, I will be telling them how she is doing, and what stage she's at in her rehab. (Inpatient rehabilitation, Occupational Therapist)

The family caregivers seemed much less aware and did not focus on these regulations. None of the family caregivers commented that patient's privacy was a possible rationale for health care providers to not share patient information with them. When family caregivers did encounter privacy regulations acting as a barrier to communication, for example, using the telephone to access hospital information from health care providers, they expressed frustration related to their lack of education regarding the necessary procedures.

#### Information sharing is difficult due to the large number of people involved

The family caregivers felt that there was a lack of continuity when they described communicating with the teams of health care providers on the units. Researchers observed that the family caregiver would express a level of comfort with a certain provider, but then due to shift work hours, would not see that provider again for almost a week. Family caregivers explained their perception that the health care providers were often uninformed either between shifts or between specialities. The family caregivers felt that this led to poorer care quality and expressed frustration related to the difficulties they experienced gathering information. Two family caregivers describe how they felt in the following passages.

I really think that, it's almost like spot, the treatment's like spotted. If there was somebody consistently around it may be better because then that's a concentration and they would know they would have consistent statements; ok now you can start walking with a walker rather than me having to ask for it down the road. Today I was talking to the head nurse and she says she would have to look at the notes in the end. So really she doesn't know and she doesn't know because she is not there…There's no cushion, there's no consistency. It is almost like spot check. (Family Caregiver)

I mean it's extremely frustrating to try and find somebody who actually knows what the situation is, to find somebody who actually if they don't know they'll find out or they'll find somebody who knows and send them to the room or they'll check the chart or they'll ask. Most people's response is well I don't know or she's on break, ok could you please let her know when she comes back from break; well I might not see her. I said then how am I supposed to find the person that knows. (Family Caregiver)

The family caregivers also had difficulties sharing information with multiple team members. They were unsure who to communicate with and when.

If CCAC knows I'm the primary person, so should the nurses by now, I shouldn't have to go and tell them because I didn't know. This is our first experience with having someone in the hospital, thank god. But that's, there was no way, I knew that this was what was required from me, that they need to have my phone number to be able to call me. So if I'm going to the CCAC I'm assuming everyone else knows it. (Family Caregiver)

The health care providers also recognised this issue. They often described that shift work made it difficult to maintain continuity within the team. They also recognised that changing health care providers made it difficult for the patient and family caregivers to communicate with them.

For consistency for the floor in that they're not looking at a different face you know every day and say you know yesterday [one health care providers] said this, well I don't know I haven't read her note yet, that sort of thing. So for that consistency I don't like it and for the consistency of the patient…it's not the most efficient. You feel like you're spending your whole first day back just trying to learn your case load again. (Acute care, Inhospital CCAC case manager)

Both the family caregiver and health care providers also recognised that it was more difficult to share information when multiple family members were involved and a single primary contact person was not identified. For example, one family caregiver explained:

I actually am probably going to call this morning and you know see what I can find out about, it's really hard when there is more than one person looking after it. Like my sister gets some information and I get some information. You know we try to share it but you know what I mean. (Family Caregiver)

#### No clear organisation or process is used to guide information sharing

The quantity and type information, as well as when it was shared, varied by patient and appeared very sporadic and arbitrary. The health care providers referred to this as a ‘team-based approach’ forcommunicating with the family caregivers. They described that each provider would collect or share small pieces of information with the family caregivers either based on the health care providers' specialty or direct questions asked by the family caregivers. They would then rely on multidisciplinary meetings, written charts or casual conversations to inform the other health care providers what information was shared. The health care providers favoured this strategy and referred to it as a multidisciplinary approach.

When [the patients] are discharged we have CCAC come in when they are involved, so we all everybody kind of talks to the family, like CCAC gets involved so it is just kind of like a whole team effort.. . I knew that it had been arranged already. I don't know by who but it had been arranged. (Inpatient rehabilitation, nurse).

While there were approaches in place to support communication strategies between health care providers, it was observed that some of these strategies were not consistently utilised. The researchers observed a white board available in each patient's room, which was meant to have the date and current care provider's name and title to keep the patient and family aware of who was working with them. However, each time the patient's room was entered by a researcher, it was observed that the whiteboard was often a week out-dated and the provider had changed many times.

The health care providers also explained that they often relied on their clinical impression while caring for the patient to determine whether they should contact the family caregivers. As well, it was common for the health care providers to give general descriptions reflecting on the importance of interacting with the family caregivers; however, when asked to describe their specific interactions within this circle of care, they often had little or no contact with the family caregivers. Conversely, family caregivers did not like this strategy for information sharing. Family caregivers perceived communication with health care providers as sporadic or spotted and that they were often receiving different information from the various health care providers.

When we used to go visit we used to get different advice as to what to do, how to do, what to take in, at different times…We went in and we took the purses in, we wore the gown and we wore the gloves first two days. The third day there was somebody else and she said do not take your purse in, so we had to leave it out. Things like that, it's like ‘ok, we've been taking it in’ so things like that were irksome. (Family Caregiver)

[The Community PT] said ‘oh you should stand up for ½ hour and then lie down, you shouldn't be walking very much’. And I said ‘[the doctor] told us that he could walk as much as he wanted around the house’…so I did not appreciate this physio contradicting not only what the physio at the hospital had told us, what [the doctor] told us. (Family Caregiver)

It was also rare for health care providers and family caregivers to have scheduled appointments. Frequently, they would describe their encounters as a ‘fluke’.

Actually her daughter phoned me I think before I had a chance to meet with her. But I was able to give her the background I had from the bullet rounds. And then by fluke I was in with her and her daughter was in…So it was just coincidental we didn't arrange to have a meeting. (Acute care, Inhospital case manager)

Health care providers and family caregivers often assumed the other group had the information they needed without directly speaking to them. For example, written information was available for family caregivers; however, it was not being used effectively. The health care providers were unaware who was responsible for distributing the information to the family resulting in the family caregivers not receiving this information until it was too late.

There is a pamphlet but I personally didn't - something about rehab pamphlet. I don't know. I don't know if she even received it, I just remember hearing them [other health care providers] talk about it. I did not give it to her because I'm still looking for this myself…Yeah it should have been. I don't know if it was done. (Inpatient rehabilitation, nurse)

There was a pamphlet, lots of write ups on his desk all the time and I would look at them…but actually saw it only today, I mean yesterday. So maybe it was there and I never saw it, but I go through things. (Family Caregiver)

## Discussion

Previous studies have identified that poor information sharing between family caregivers and health care providers has a negative impact on transitional care for older patients [17,28,38,39]. In order to develop targeted interventions, it was essential to further explore why these groups have difficulties communicating. By interviewing both family caregivers and health care providers as they interacted in caring for the same patient, we were able to explore their immediate perspectives on information sharing during in-hospital and transitional care. Our findings described the groups' attitude towards communicating, as well as identified five issues that negatively influenced information sharing between family caregivers and health care providers that may be potential targets for future intervention.

We found that both family caregivers and health care providers undoubtedly recognised the value of including family caregivers in the circle of care. This viewpoint corresponds with the current position in the literature which supports collaborative interactions between these groups in order to achieve optimal care for ill patients [34,65,66]. Our findings support that these groups currently understand the benefits of communicating. Thus, in order to develop effective interventions, strategies to promote information sharing should focus on changing actual behaviours rather than purely encouraging collaborative attitudes. Having policies and procedures formed around discharge planning earlier in the process will also provide opportunities for smoother transitional care and for understanding expectations and the feasibility of care plans.

The family caregivers and the health care providers both described a non-standardised process when they recounted the current approach for sharing information. The health care providers positively reflected on this strategy and often associated it with working as a team, while the family caregivers perceived the lack of standardisation as disorganised and poor quality care. One possible reason for these opposing perceptions is that health care providers are less affected by missing information, as they have additional strategies in place to stay informed, such as frequent multidisciplinary meetings and updated medical charts; whereas the family caregivers currently do not have access to these resources. Establishing similar strategies that engage both health care providers and family caregivers may facilitate information sharing between these groups. Wittenberg-Lyles and colleagues [67,68] used video-conferencing (tele-health) to involve family caregivers of hospice patients in bi-weekly multidisciplinary meetings. They found that meetings that included family caregivers had better team outcomes, more emphasis on biomedical education and relationship building between family caregivers and health care providers, more inter-disciplinary care plans and increased socio-emotional communication with social workers and chaplains [67,68]. Various modes of health information technology have also been successfully used to assist communication between health care providers and family caregivers of paediatric patients [69]. Appealing approaches included Internet, video-conferencing, and/or short messaging services; these achieved outcomes such as establishing continuity of care, addressing time constraints and bridging geographical barriers [69]. Allowing patients and family caregivers access to medical records is also being considered in Canada and other countries as a strategy for keeping these groups informed [70].

A common argument against more standardised approaches to care is that older patients represent a very heterogeneous group with individualised needs. Consequently, in this population determining effective principles, as opposed to stringent clinical practice guidelines, to be used as a framework for supporting more comprehensive information sharing during care transitions may be most effective. A similar approach has been suggested in cancer care in order to develop clear strategies within which to work with both patient and family members [71]. For example, Conatser [72] acknowledged patient heterogeneity in this population and proposed a general list of the types of information that must be exchanged between family caregivers and health care providers through the treatment course for cancer. Information sharing during care transitions may benefit from future research to develop a similar list.

In the current environment where there are few structured approaches for keeping family caregivers informed, our participants described that family caregivers are largely responsible for seeking information from health care providers. Interestingly, the health care providers felt this pattern was appropriate and supported information sharing; while the family caregivers expressed frustration and described needing to approach health care providers and maintaining communication as barriers for information sharing. Previous studies have also identified that family caregivers are often required to instigate and direct communication with health care providers [12,25,43,73]. A foreseeable problem with this approach is that family caregivers needs and roles change throughout care transitions [74,75]. One study that focused on care transitions from hospital to home, described that family caregivers were less aware of their information needs while the patient was in the hospital, however, after discharge the family caregivers realised that they were missing essential knowledge required to carry out their role [74]. This demonstrates that there are potential harms of relying on family caregivers to elicit information from health care providers based on their perceived needs.

When family caregivers and health care providers more specifically described their process for sharing information, both groups focused nearly exclusively on receiving information from the other party. Though there is little research on specific components of effective communication between family caregivers and health care providers, previous studies regarding doctor–patient communication advised that two-way interactions are necessary to promote successful information sharing [76]. To establish effective communication between these family caregivers and health care providers that has the potential to lead to positive patient outcomes, it is essential that future interventions focus on promoting mutually beneficial relationships between health care providers and family caregivers [77].

## Limitations

A number of limitations exist within this current study. This research project focused solely on the experiences between family caregivers and health care providers. This may have biased the findings by not taking into consideration the perspectives of patients. In some instances, the patient's experience may have reinforced communication barriers and challenges.

As our study population included a greater fraction of Community Care Access Centre case managers relative to other health care providers, our findings may disproportionally represent the views of this sub-group. Though the scope of their experiences tended to be limited to issues surrounding discharge planning and community care, this group is highly involved in transitional care for older hip fracture patients.

Second, patients who were not able to provide informed consent were excluded from the study, and therefore, interviews were not conducted with family caregivers who acted as substitute decision-makers. Thus, the findings of our study may not reflect additional issues relevant to this group of family caregivers. As well, a high proportion of family caregivers who were interviewed had backgrounds working as health care providers. Their previous work experience and added knowledge about the health care system may have influenced how they communicate with health care providers across the continuum of care. Regardless, issues of information exchange were still found within this study. These issues may be more pronounced for family caregivers who do not have work-related histories with the health care system.

Lastly, one site (the city of Waterloo) was used to conduct this study. It is possible that other regions have different infrastructure surrounding transitional care, therefore, some of the issues found within the study may become non-existent or enhanced in different sized cities. This study was conducted in a mid-sized city with both urban and rural townships, which assists in making the findings more generalisable in both larger-scale and smaller-scale cities.

## Conclusions

This study has identified specific barriers that lead to poor information sharing between family caregivers and health care providers. Additional research is required to investigate potential interventions to address these barriers. Interventions to improve information sharing could include encouraging communication with family caregivers as standard care practice, educational strategies and more effective use of health information systems and technologies. Creating policies to include family caregivers as an important part of the circle of care and inclusion of families in the development of discharge and care plans is also essential. Developing better partnerships between family caregivers and health care providers that facilitate communication and information sharing during care transitions is an ambitious goal that will take considerable effort and time to realise. Developing supplementary resources for family caregivers such as caregiving websites [78] and patient–caregiver portals [79] may also be helpful to address the needs of family caregivers in the interim. Utilising these tools is an efficient method of sharing information between all parties involved in the patient's care. Encouraging these tools as part of a standard practice of care may enhance information sharing, and thus reduce the frustrations associated with poor communication and lead to increases in the quality of care provided to complex patients.

## Figures and Tables

**Table 1.**
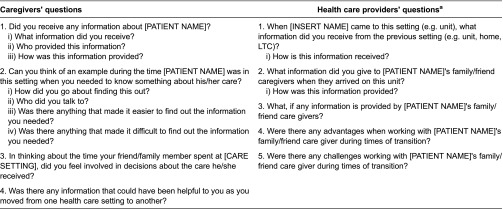
Sample interview questions directed towards caregivers and health care providers

^a^For a full list of questions from the health care providers' interview, guide please see Toscan et al. [58] Appendix A.

**Table 2.**
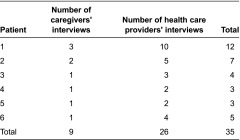
Number of interviews in each care network

**Table 3.**
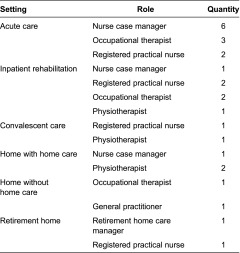
Health care providers' setting and role

**Table 4.**
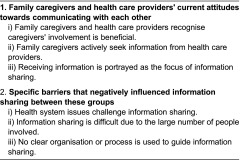
Summary of themes and sub-themes
